# The neutrophil to lymphocyte ratio is an independent predictor for severe COVID-19: Evidence from a multicenter case-control study and meta-analyses

**DOI:** 10.1007/s00508-021-01917-9

**Published:** 2021-08-03

**Authors:** Jiangli Cheng, Aijia Ma, Jing Yang, Meiling Dong, Xuelian Liao, Yan Kang

**Affiliations:** Department of Critical Care Medicine, West China Hospital of Sichuan University, No. 37, Guoxue Alley, 610041 Chengdu, Sichuan Province, China

**Keywords:** Coronavirus disease 2019, Neutrophil, Lymphocyte, Risk factor, Severity

## Abstract

**Purpose:**

The aim of this study was to determine whether the neutrophil to lymphocyte ratio (NLR) can predict severe Coronavirus disease 2019 (COVID-19).

**Patients and methods:**

A multicenter case-control study was conducted to investigate whether the NLR can help predict the severity of COVID-19. Patients confirmed to have COVID-19 between 16 January 2020 and 15 March 2020 were enrolled. Furthermore, meta-analyses were conducted based on both previous studies and our case-control study.

**Results:**

In the case-control study, 213 patients (severe: 81) were included. The results suggested that the NLR was an independent risk factor (odds ratio [OR], 1.155, 95% confidence interval [95% CI]: 1.043–1.279, *P* = 0.006) and a great predictor (the area under the ROC curve was 0.728, 95% CI: 0.656–0.800) for severe COVID-19. In total, 18 datasets from 16 studies combined with our case-control study (severe: 1211; non-severe: 5838) were included in the meta-analyses and the results showed that the NLR of the severe COVID-19 group was significantly higher than that of the non-severe group (SMD = 1.10, 95% CI: 0.90–1.31, *P* < 0.001). Based on the 2 × 2 data from 6 studies, the SROC of NLR for predicting severe COVID-19 was 0.802, with a sensitivity of 0.67 (95% CI: 0.61–0.72) and a specificity of 0.75 (95% CI: 0.73–0.78).

**Conclusion:**

Based on a multicenter case-control study and a meta-analysis, we found that the initial NLR was a great predictor of severe COVID-19.

**Supplementary Information:**

The online version of this article (10.1007/s00508-021-01917-9) contains supplementary material, which is available to authorized users.

## Introduction

The current pandemic of coronavirus disease 2019 (COVID-19) has spread rapidly all over the world [1]. To date, more than 11.8 million people worldwide have been diagnosed with COVID-19: among them 20–30% have developed into severe COVID-19 [2]. According to previous studies, the mortality rate of severe COVID-19 patients is approximately 20 times higher than that of non-severe COVID-19 patients, up to 61.5% [3, 4]. A sudden transition to severe COVID-19 is common for patients with mild symptoms [5]. Clinical deterioration will lead to the consumption of medical resources, intensive care beds, ventilators, and adverse outcomes of the patients. Hence, it is very important to identify an efficient predictor for the early identification and targeting of potentially severe patients to improve the clinical outcomes of patients with COVID-19.

COVID-19 is characterized by uncontrolled inflammatory storms [6]. The neutrophil to lymphocyte ratio (NLR), a routinely measured inflammatory biomarker, reflects the immune status of the human defence system against infection. A higher NLR, which results in an increased neutrophil or/and decreased lymphocyte count, might indicate that the patient had severe inflammatory progression [7]. Some studies have reported that the NLR could be useful for the diagnosis of sepsis and might be a good predictor for a poor prognosis of acute respiratory distress syndrome, which has the same disease characteristics as COVID-19, to some extent [8–10]. Previous studies of COVID-19 have noted the predictive power of the NLR for clinical deterioration and mortality among COVID-19 patients [11–13]; however, whether NLR can predict severe COVID-19 is still controversial. Thus, a multicenter case-control study and meta-analysis was conducted to estimate the potential predictive value of the NLR for severe COVID-19.

## Material and methods

### Case-control study

#### Study design and participants

In total, 213 patients (3 patients were excluded due to missing NLR data at admission) with confirmed COVID-19 from 21 hospitals in Sichuan Province between 16 January 2020 and 15 March 2020 were included in the analysis: among them, 81 patients were defined as having severe COVID-19. The definitions of COVID-19 and severe COVID-19 were described in a previously published article [14]. The protocol was approved by the Ethics Committee of the West China Hospital of Sichuan University and the participating hospitals, and informed consent was obtained. The study was registered and the registration number was ChiCTR2000029758.

#### Data collection

We conducted the study based on the electronic data capture and analysis system for each included patient. Demographic characteristics, comorbidities, symptoms, signs and laboratory findings at admission were collected. The NLR is the ratio of neutrophil count to lymphocyte count, and platelet to lymphocyte ratio (PLR) is the ratio of platelet count to lymphocyte count.

#### Statistical analysis

Categorical variables are expressed as numbers and percentages, continuous normally distributed variables are expressed as the mean and standard deviation, and continuous skewness-distributed variables are expressed as the median and interquartile range. The χ^2^-test, t‑test and Mann–Whitney U test were used to compare the differences between severe and non-severe COVID-19 groups. A 2-tailed *P* value lower than 0.05 was considered to be statistically significant. Variables found to be statistically significant in univariate analysis were included in the multivariate logistic regression analysis to identify the independent risk factors for severe COVID-19. The odds ratio (OR) and its 95% confidence interval (CI) were used as binary parameters. The Cochran-Armitage test for trend and the area under the receiver operating characteristic (ROC) curve were used to analyze the linear relationship of NLR level with the rate of severe COVID-19 and the ability of NLR to predict severe COVID-19, respectively.

### Meta-analyses

This meta-analysis conformed to the preferred reporting items for system review and meta-analysis (PRISMA) statement ([15]; Additional file 1).

#### Data search

PubMed, EMBASE, Web of Science, MedRxiv and BioRxiv, the WanFang database and the China National Knowledge Infrastructure (CNKI) were searched through inception to May 2020. The search terms of ‘neutrophil-to-lymphocyte ratio’ OR ‘NLR’ and ‘COVID-19’ were used to identify studies that reported the relationship between NRL and the severity of COVID-19. The reference lists of the included studies were examined to identify any other potentially qualified studies.

#### Study selection

Two reviewers reviewed the titles and abstracts independently to determine whether they needed full-text review according to the following inclusion and exclusion criteria. After that the full-text review of the studies was completed to identify the articles included by the same two reviewers. When the opinions were inconsistent, the question was discussed and determined by a third reviewer.

##### Inclusion and exclusion criteria

Studies meeting the following criteria were included:

1. Patients diagnosed with COVID-19 and could be divided into severe and non-severe COVID-19 groups.
2. The data of NLR were available and be compared between the severe and non-severe COVID-19 groups.

Studies with the following criteria were excluded:

1. Articles not written in English or Chinese
2. Insufficient data for analysis.
3. Repeated published data.
4. Reviews, systematic reviews, meta-analyses, guidelines, study protocols, case reports, letters, correspondences, comments or editorials.

#### Data extraction

Data were extracted using the predesigned data collection table. Two reviewers extracted data from all included studies independently. The collected data included author, year, study design, study location, study samples, NLR values, age and sex. When the opinions were inconsistent, the question was discussed and determined by a third reviewer.

#### Assessment of study quality

Studies were evaluated by the Newcastle–Ottawa assessment tool, for which the maximum score was 9 points and a score of 7 or greater was considered to be a low risk of bias [16]. Two reviewers assessed the risk of bias of all of the included studies independently; when opinions were inconsistent, the question was discussed and determined by a third reviewer.

#### Data synthesis and analysis

The software Stata version 16.0 (STATA Corporation, College Station, TX, USA) and Meta-Disc, version 1.4 (Unit of Clinical Biostatistics team of the Ramón y Cajal Hospital, Madrid, Spain) were used to conduct the statistical analysis. The median and interquartile range were transformed into mean and standard deviation based on a previously proven formula; [17, 18]. Standardized mean differences (SMDs) and corresponding 95% confidence intervals (CIs) were used to evaluate the differences in NLR between the severe COVID-19 and non-severe COVID-19 groups. Heterogeneity among the studies was evaluated using Cochran’s Q test and the I^2^ statistic. An I^2^ index of 50% or greater and a *p*-value less than 0.1 were taken to indicate significant statistical heterogeneity. When statistical heterogeneity existed, a random effects model was used. Publication bias was assessed by Egger’s and Begg’s tests [19]. Subgroup analyses were performed according to the mean age (> 50 years or ≤ 50 years) and study location (the study included patients in Hubei or not).To further analyze the predictive power of the NLR in severe COVID-19, 2 × 2 data points were obtained from the studies. The area under summary receiver operating characteristic (SROC), Q index, sensitivity and specificity with 95% CI were calculated.

## Results

### Case-control study

A total of 213 patients were enrolled in the final analysis, with 81 severe COVID-19 patients. Among the included patients, 94 (44.1%) patients were female; the median age was 47 years (33, 59 years); fever was the most common symptom before admission, and diabetes was the most common comorbidity. Age, body mass index, mean arterial pressure, heart rate, respiratory rate, neutrophil count, NLR, PLR, aspartate aminotransferase and alanine aminotransferase were higher in the severe COVID-19 group than in the non-severe group, and pulse oxygen saturation and lymphocyte count were lower in the severe COVID-19 group. Severe COVID-19 patients were more likely to have fever, sputum production, fatigue, dyspnea, diabetes, hypertension and chronic pulmonary disease (see Table 1).

**Table 1:** Characteristics of the study population

Variables	Total (*n* = 213)	Mild COVID-19 (*n* = 132)	Severe COVID-19 (*n* = 81)	*P*
*Characteristics*				
Age, years	47 (33, 59)	43 (31, 54)	50 (39, 65)	0.000
Gender/case (%)				0.102
Male	119 (55.9%)	68 (51.5%)	51 (63%)	–
Female	94 (44.1%)	64 (48.5%)	30 (37%)	–
BMI/(kg/m^2^)	23.0 (21.1, 25.9)	22.6 (20.8, 24.5)	24.0 (21.5, 27.3)	0.013
*Chronic medical illness/case (%)*				
Diabetes	25 (11.7%)	7 (5.3%)	18 (22.2%)	0.000
Hypertension	22 (10.3%)	7 (5.3%)	15 (18.5%)	0.002
Chronic pulmonary disease	15 (7.0%)	4 (3%)	11 (13.6%)	0.003
*Signs and symptoms*				
Fever (> 37.3℃)	150 (70.4%)	81 (61.4%)	69 (85.2%)	0.000
Dry cough/case (%)	100 (46.9%)	60 (45.5%)	40 (49.4%)	0.577
Sputum production/case (%)	73 (34.3%)	35 (26.5%)	38 (46.9%)	0.002
Fatigue/case (%)	59 (27.7%)	29 (22%)	30 (37%)	0.017
Dyspnea/case (%)	37 (17.4%)	12 (9.1%)	25 (30.9%)	0.000
Mean arterial pressure/mm Hg	94.67 (87.17, 101.67)	93 (85.5, 99.9)	96 (89.7, 106.2)	0.012
Heart rate/(beats/min)	90 (80, 98)	88 (80, 96)	90 (86, 101.5)	0.031
Respiratory rate/(breaths/min)	20 (20, 21)	20 (20, 21)	21 (20, 23)	0.000
Pulse oxygen saturation/%	97 (95, 98)	98 (96, 98)	95.5 (91.75, 97)	0.000
*Blood laboratory findings*				
White blood cell count/10^9/L	5.41 (4.08, 7.08)	5.30 (4.14, 6.92)	5.73 (4.05, 7.61)	0.153
Neutrophil count/10^9/L	3.65 (2.63, 5.24)	3.38 (2.56, 4.72)	4.37 (2.91, 6.40)	0.001
Lymphocyte count/10^9/L	0.99 (0.67, 1.51)	1.25 (0.83, 1.68)	0.73 (0.51, 1.05)	0.000
NLR	3.28 (2.22, 5.80)	2.88 (2.07, 4.16)	5.41 (2.90, 9.87)	0.000
Platelet count/10^9/L	168 (132.5, 216.5)	180.5 (140.25, 224.25)	151 (126.5, 205)	0.064
PLR	169.86 (112.04, 258.20)	139.91 (107.09, 218.77)	206.3 (141.1, 338.68)	0.000
Hemoglobin/(g/L)	138 (125, 150)	138 (126, 150)	137 (123, 150)	0.319
Total bilirubin/(µmol/L)	8.85 (5.9, 14.5)	8.4 (5.6, 14)	9.2 (6.35, 15.2)	0.228
AST/(IU/L)	29 (23, 38.5)	28 (22, 35)	32 (25, 44.68)	0.006
ALT/(IU/L)	26 (16, 41)	22 (16, 39)	30 (17.5, 45.25)	0.060
Creatinine/(µmol/L)	67.8 (53.85, 78.68)	66 (52.48, 76)	69.75 (54.63, 80)	0.064
Lactic acid/(mmol/L)	1.6 (1.2, 2.1)	1.6 (1.2, 1.9)	1.62 (1.2, 2.3)	0.383

Data are presented as interquartile range or number (percentage)

*COVID-19* Coronavirus disease 2019, *BMI* body mass index, *NLR* neutrophil-to-lymphocyte ratio, *PLR* platelet-to-lymphocyte ratio, *AST* aspartate aminotransferase, *ALT* alanine aminotransferase

For all patients, the median NLR was 3.28 (2.22, 5.80), with 2.88 (2.07, 4.16) in the non-severe group and 5.41 (2.90, 9.87) in the severe COVID-19 group. Variables found to be statistically significant (*P* <0.05) in univariate analysis were included in the multivariate logistic regression analysis to identify the independent risk factors for severe COVID-19. After adjustment for confounders, the NLR was found to be an independent risk factor for severe COVID-19 (OR, 1.155, 95% CI, 1.043–1.279, *P* = 0.006).

**Fig. 1:**
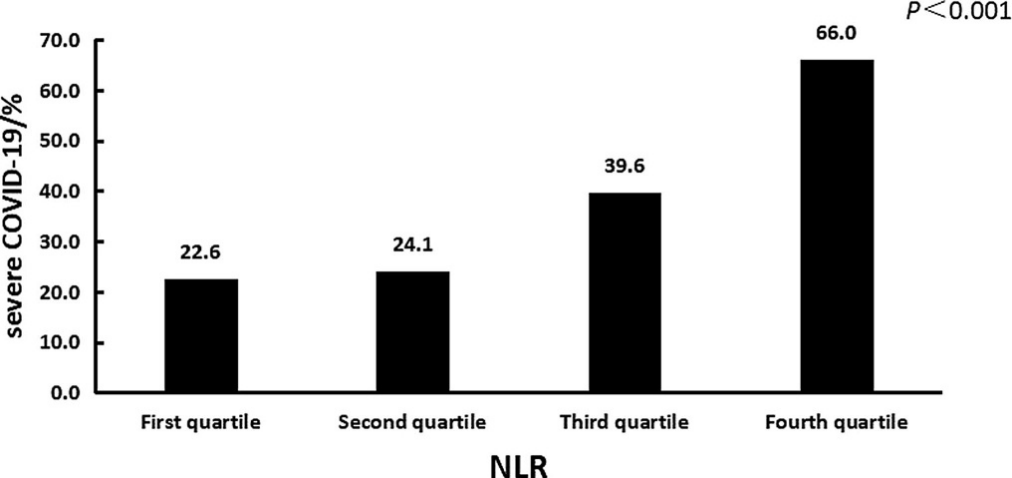
The occurrence rate of severe COVID-19 of each quartile of NLR

The patients were divided into 4 groups according to the interquartile NLR, they were less than 2.22, 2.22–3.28, 3.28–5.80 and greater than 5.80. The occurrence rates of severe COVID-19 in each quartile group were calculated, and were 22.6%, 24.1%, 39.6% and 66% in the first quartile, second quartile, third quartile and fourth quartile, respectively. Based on the statistical analysis result of the Cochran-Armitage test for trend, there was a linear relationship between the NLR level and the occurrence rate of severe COVID-19, which visibly increased with the increase in NLR (*P* <0.001). The result is described in Fig. 1.

**Fig. 2:**
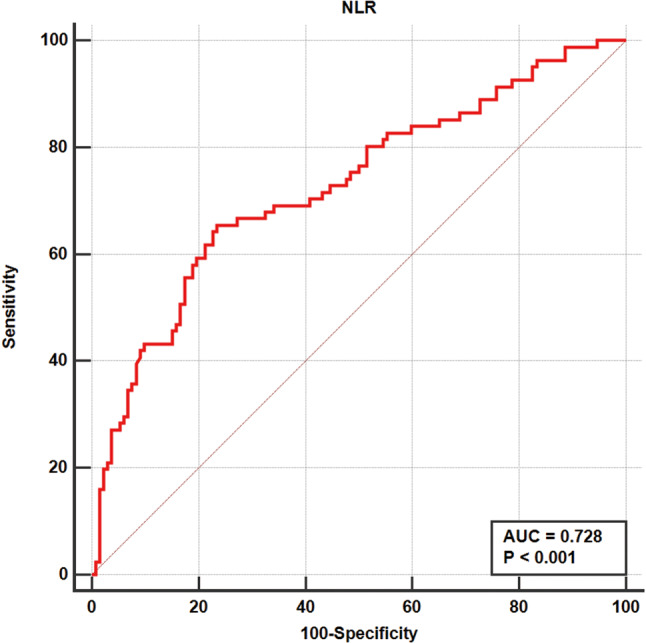
The ROC curve in predicting severe COVID-19 of NLR

An ROC curve analysis was performed to analyze the ability of the NLR to predict severe COVID-19. The area under the ROC curve was 0.728 (95% CI, 0.656–0.800). The optimal cut-off value was 4.184 with a sensitivity of 0.654 and a specificity of 0.765. The result is described in Fig. 2.

Multivariable analysis found that age (*P* <0.001), body mass index (*P* = 0.038), fever (*P* = 0.012), sputum production (*P* = 0.019), respiratory rate (*P* = 0.004), pulse oxygen saturation (*P* = 0.001) and NLR (*P* = 0.006) contributed independently to severe COVID-19. The result is described in Additional file 2.

### Meta-analyses

#### Literature search and study selection

**Fig. 3:**
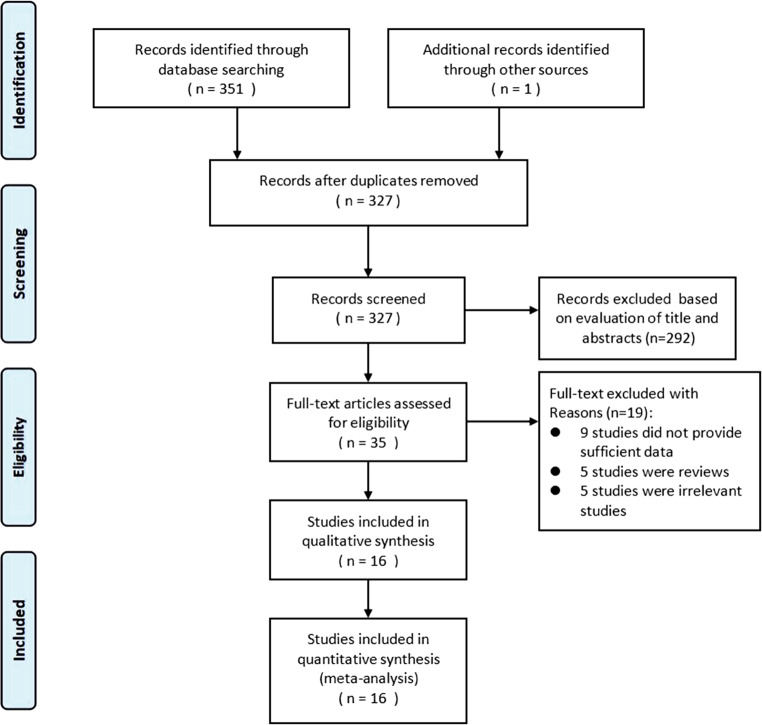
The flow of citations reviewed and studies included

Fig. 3 shows the work flow of the citations reviewed and the studies included. Our search strategy yielded 327 citations after de-duplication, and 35 potential citations were full-text reviewed. Finally, 16 citations were included. The study from Yabing Guo and Wenhua Liang reported 2 sets of data. Therefore, 19 datasets were included in the quantitative synthesis (18 datasets from 16 studies combined with our case-control study) [6, 20–34].

#### Study characteristics

A total of 7049 (6836 patients in the previous study and 213 patients in our study) patients were included, among whom 1211 (1130 patients in the previous study and 81 patients in our study) patients had severe COVID-19. The sample size of the individual studies ranged from 63 subjects to 1590 subjects. Only one study was from Britain, and all other studies were from China, with 8 studies included patients in Hubei. One of these studies was prospectively conducted, and all of the others were retrospectively. All studies reported patient ages, and the mean age of the patients in 5 studies was greater than 50 years old (see Table 2).

**Table 2:** Main characteristics of the included studies in the meta-analysis

Author	Study design	Study location	Samples	NLR value	Mean age (years)	Sex (female %)
Qin et al. 2020	Retrospective	Hubei, China	Non-severe: 166 Severe: 286	Non-severe: 3.2 (1.8, 4.9) severe: 5.5 (3.3, 10.0)	57.3	48.0%
Gong et al. 2020	Retrospective	Guangdong and Hubei, China	Non-severe: 161 Severe: 28	Non-severe: 1.9 (1.4, 2.9) Severe: 3.7 (2.0, 6.7)	49.2	53.4%
Liu et al. 2020	Prospective	Beijing, China	Non-severe: 44 Severe: 17	Non-severe: 2.2 (1.4, 3.1) Severe: 3.6 (2.5, 5.4)	42.5	49.2%
Yang et al. 2020	Retrospective	Zhejiang, China	Non-severe: 69 Severe: 24	Non-severe: 4.8 ± 3.5 Severe: 20.7 ± 24.1	46.4	39.8%
Zhang et al. 2020	Retrospective	Hubei, China	Mild: 84 Severe: 31	Mild: 2.28 ± 1.29 Severe: 7.58 ± 7.04	49.5	57.4%
Guo et al. 2020	Retrospective	Guangdong, China	Non-severe: 753 Severe: 65	Non-severe: 1.95 (1.36, 2.95) Severe: 3.34 (2.22–6.22)	43.4	51.6%
Guo et al. 2020	Retrospective	Hubei, China	Non-severe: 282 Severe: 38	Non-severe: 2.5 (1.68, 4.12) Severe: 4.48 (2.06, 8.49)	55.6	50%
Feng et al. 2020	Retrospective	Hunan, China	Non-severe: 126 Severe: 15	Non-severe: 3.4 (1.9, 3.9) Severe: 5.2 (3.1, 6.9)	44.4	48.9%
Chen et al. 2020	Retrospective	Hunan, China	Mild: 29 Moderate: 212 Severe: 50	Mild: 3.07 (2.13, 6.1) Moderate: 2.3 (1.67, 3.24) Severe: 3.74 (2.16, 6.54)	46.4	50.2%
Wang et al. 2020	Retrospective	Multi-center, China	Mild: 86 Ordinary: 486 Severe: 63	Mild: 2.73 ± 2.28 Ordinary: 3.58 ± 3.07 Severe: 9.38 ± 10.52	44.9	48%
Huang et al. 2020	Retrospective	Hunan, China	Non-severe: 92 Severe: 29	Non-severe: 2.55 ± 1.38 Severe: 4.4 ± 2.7	45.1	47.1%
Xia et al. 2020	Retrospective	Hubei, China	Moderate: 32 Severe: 31	Moderate: 2.89 (1.77, 5.55) Severe: 8.78 (5.77, 25.10)	63.4	47.6%
Chen et al. 2020	Retrospective	Chongqing, Chian	Non-severe: 108 Severe: 31	Non-severe: 3.19 (2.13, 4.73) Severe: 4.07 (2.86, 6.90)	45.3	45.3%
Pan et al. 2020	Retrospective	Hubei, China	Non-severe: 96 Severe: 16	Non-severe: 4.74 (2.5, 8.75) Severe: 9.25 (5.95, 11.57)	61.3	62.7%
Liang et al. 2020	Retrospective	Multi-center, China	Nonsevere: 1459 Severe: 131	Non-severe: 4.4 ± 3.8 Severe: 12.7 ± 12.4	48.9	42.7%
Liang et al. 2020	Retrospective	Multi-center, China	Non-severe: 642 Severe: 87	Non-severe: 4.3 ± 3.8 Severe: 17.1 ± 20	48.2	/
Ewan et al. 2020	Retrospective	Multi-center, Britain	No ICU/death: 393 ICU/death: 159	No ICU/death: 5.81 ± 4.22 ICU/death: 8.58 ± 6.26	67	45.1%
Huang et al. 2020	Retrospective	Shanghai, China	Nonsevere: 386 Severe: 29	Non-severe: 2.67 (1.76, 3.42) Severe: 4.16 (3.14, 14.65)	45.1	47.7%
Cheng et al. 2021	Retrospective	Sichuan, China	Mild: 132 Severe: 81	Mild: 2.88 (2.07, 4.16) Severe: 5.41(2.90, 9.87)	46.9	44.1%

Data are presented as interquartile range or number (percentage)

*NLR* neutrophil-to-lymphocyte ratio, *ICU* intensive care unit

#### Risk of bias of the included studies

The Newcastle–Ottawa assessment tool was used to evaluate the quality of the included studies. All studies were considered to have a low risk of bias (Additional file 3). Among the included studies, no significant publication bias was found (Egger’s test: *P* = 0.924; Begg’s test: *P* = 0.889; Additional file 4).

#### Association of NLR with the severity of COVID-19

**Fig. 4:**
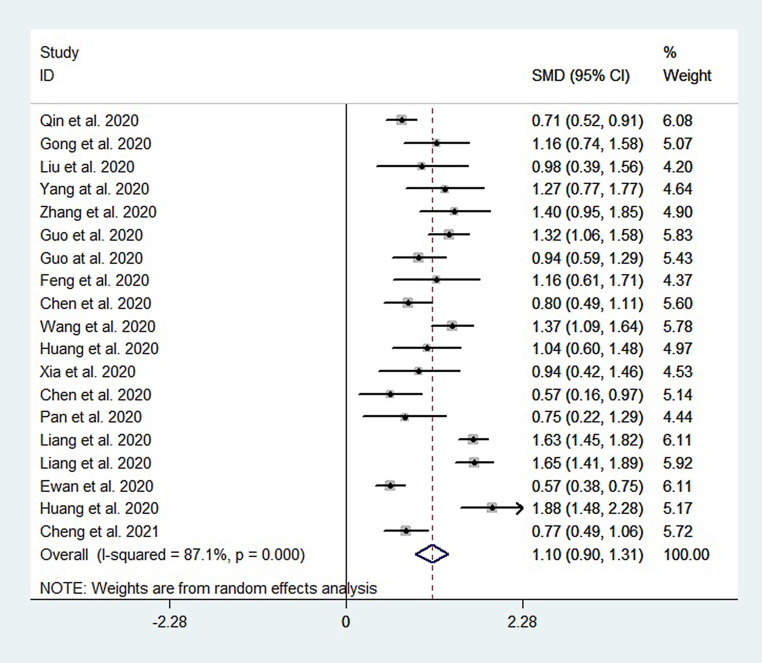
Forest plots of NLR between severe COVID-19 and non-severe COVID-19

A total of 7049 patients from 19 datasets were analyzed. The pooled results showed that NLR were higher in patients with severe COVID-19 than in those with non-severe COVID-19 (SMD = 1.10, 95% CI: 0.90–1.31, *P* < 0.001). High heterogeneity existed (I^2^ = 87.1%, *P* < 0.001, a random-effects model was used) (see Fig. 4).

#### Subgroup analyses

To determine other parameters that might affect the predictive power of NLR for severe COVID-19, subgroup analyses were conducted according to the mean age (> 50 years or ≤ 50 years) and study location (the study included patients from Hubei or not). The results showed that the mean age and the study location did not affect the predictive power of the NLR for severe COVID-19 (see Table 3 and Additional file 5).

**Table 3:** Results of subgroup meta-analysis for predicting severe COVID-19 of NLR

Variables	*N*^a^	Case^b^	Pooled data		Heterogeneity	
			HR (95%CI)	*P*	I2	Ph
NLR						
Severe vs. non-severe	19	7049	1.104 (0.900, 1.308)	**<** **0.001**	87.1%	**<** **0.001**
By mean age						
> 50 years	5	1499	0.706 (0.569, 0.842)	**<** **0.001**	14.2%	0.324
≤ 50 years	14	5550	1.228 (1.022, 1.434)	**<** **0.001**	80.7%	**<** **0.001**
By study location						
Hubei	8	3570	1.163 (0.840, 1.490)	**<** **0.001**	89.0%	**<** **0.001**
Other	11	3479	1.062 (0.801, 1.312)	**<** **0.001**	83.8%	**<** **0.001**

*SMD* standardized mean difference, *95% CI* 95% confidence interval, *P* : *p* value of pooled SMD, *I*^*2*^ value of Higgins I-squared statistics, *Ph p* value of heterogeneity test

^a^ Numbers of studies included in the meta-analysis.

^b^ Number of patients of included studies.

#### SROC

**Fig. 5:**
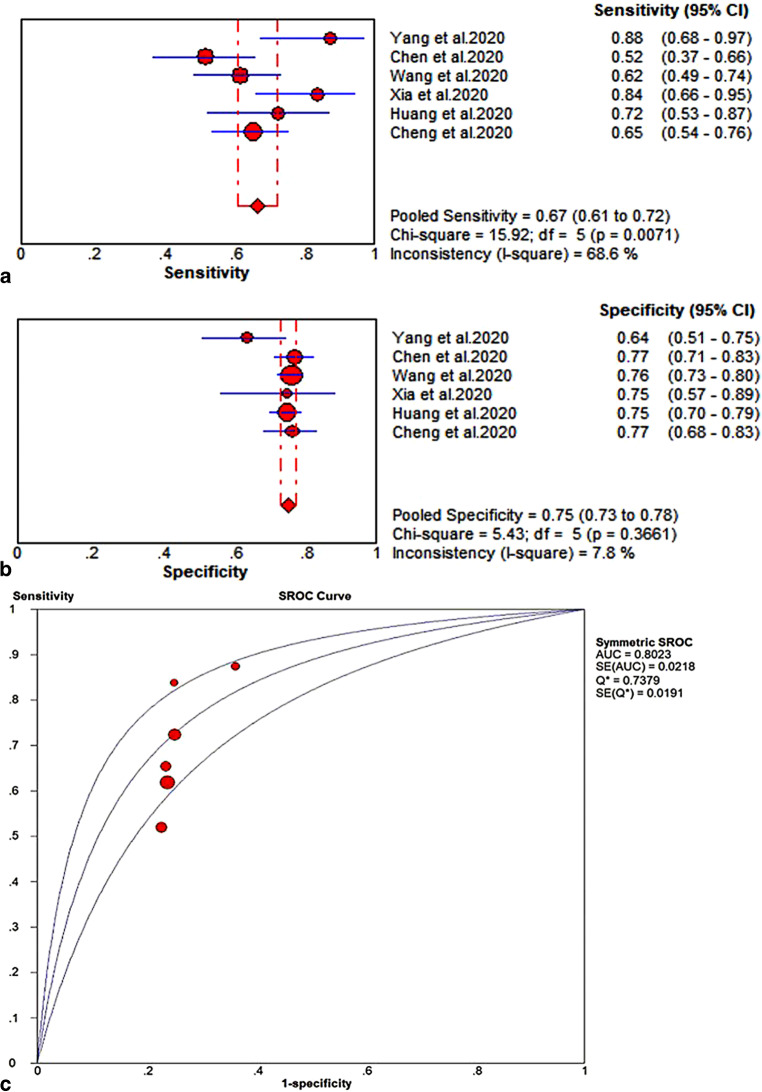
The SROC of NLR in predicting severe COVID-19. **a** Forest plot of the sensitivity; **b** Forest plot of the specificity; **c** the area under SROC and Q index

The 2 × 2 data were obtained from 6 studies. In total, 1698 patients, including 278 severe COVID-19 patients, were included in the final analysis (see Additional file 6). The area under the SROC was 0.802, and the Q index was 0.738, with a sensitivity of 0.67 (95% CI: 0.61–0.72) and a specificity of 0.75 (95% CI: 0.73–0.78) (see Fig. 5).

## Discussion

Currently, COVID-19 has spread rapidly around the world. Dysregulation of the immune response leads to clinical deterioration of patients with COVID-19, which results in increased mortality and consumption of medical resources. Early prediction and intervention for severe COVID-19 is essential to improve the prognosis of COVID-19 patients. The NLR represents the balance between neutrophil and lymphocyte levels in the body, and it has a higher sensitivity than neutrophils and lymphocytes alone to indicate the state of systemic inflammation [35, 36]. Through a case-control study and a comprehensive meta-analysis based on previous evidence and our findings, we found that the value of NLR was higher in severe COVID-19 group than in the non-severe COVID-19 group. The risk of severe COVID-19 increased with increasing NLR. The initial NLR was a strong predictor of severe COVID-19. To our knowledge, our meta-analysis is the largest study to investigate whether the NLR could help predict the severity of COVID-19 early in the disease course.

The results of our analysis are consistent with previous studies. Previous meta-analyses that included 3 studies found that NLR was positively correlated with the severity of COVID-19 [37]. This result was also consistent with another meta-analysis that included 5 studies [38]. A difference in the initial NLR was also found between patients with and without COVID-19. Wang et al. discovered that the initial NLR was a great diagnostic indicator to distinguish COVID-19 [32]. Studies have reported that the NLR could be considered as an independent biomarker of a poor prognosis in patients with COVID-19. Liu et al. showed that the NLR was an independent risk factor for mortality in COVID-19 patients [21]; however, only two studies focused on mortality and NLR for COVID-19, which is too few to perform a meta-analysis. To further confirm the significance value of the NLR in the diagnostics, prediction of disease deterioration and clinical outcomes of COVID-19 patients, more studies are necessary.

There is a large body of evidence to suggest that age plays an important role in the disease development and prognosis of COVID-19. Study of Liu et al. declared that patients with aged greater than 50 years and with an NLR greater than 3.13 were more likely to develop severe COVID-19, and they suggested that these patients should be sent to the ICU as earlier as possible [11]. While comparing the analysis results from previously published articles, patient location caught our attention, and the clinical characteristics and prognosis were different among different districts [14]. Therefore, we performed a subgroup analysis according to the mean age (> 50 years or ≤ 50 years) and study location (the study included patients in Hubei or not). The analysis showed that the mean age and the study location did not affect the predictive power of NLR for severe COVID-19. The transprovincial flow of patients should be taken into consideration when interpreting the results.

Several limitations exist in this study. First, the case-control study was conducted retrospectively, which has inherent biases although the data in the electronic data capture and analysis system were collected prospectively. Second, there were a few studies reporting the NLR of COVID-19 patients who could not be included in the final analyses due to insufficient data. Third, noticeable heterogeneity existed among studies included in the meta-analyses and was not fully eliminated, which might make the results less powerful.

## Conclusion

The initial NLR was a great predictor of severe COVID-19 and could help clinicians identify of potentially critical patients early and allocate of critical resources.

## Supplementary Information

Additional file 1.doc: PRISMA 2009 checklist of the meta-analyses

Additional file 2.doc: Multivariate logistic regression analysis results for severe COVID-19 of the case-control study.

Additional file 3.doc: Newcastle—Ottawa quality assessment scale for case-control study from the meta-analyses

Additional file 4.jpg: Begg’s and Egger’s tests of meta-analyses.

Additional file 5.jpg: The forest plots of subgroup analyses of the meta-analyses. (a) by mean age (> 50 or ≤ 50 years); (b) by study location (Hubei or other).

Additional file 6.doc: The 2 × 2 data of NLR in predicting severe COVID-19 of the meta-analyses

Additional file 7.doc: The information of hospitals and investigators participated in the case-control study.

## List of abbreviations

ALT: Alanine aminotransferase
AST: Aspartate aminotransferase
COVID-19: Coronavirus disease 2019
NLR: Neutrophil to lymphocyte ratio
PLR: Platelet to lymphocyte ratio
ROC: Receiver operating characteristic
SMD: Standardized mean difference
SROC: Summary receiver operating characteristic
