# Supplementary material for: The neutrophil to lymphocyte ratio is an independent predictor for severe COVID-19: Evidence from a multicenter case-control study and meta-analyses
Source: Wien Klin Wochenschr. 2021 Aug 3;133(17-18):882–91. doi: 10.1007/s00508-021-01917-9 (PMC8329905; doi:10.1007/s00508-021-01917-9)
Supplement: Supplementary file 2 — Additional file 2.doc: Multivariate logistic regression analysis results for severe COVID-19 of the case-control study. [file 508_2021_1917_MOESM2_ESM.doc]

Supplemental Table 1. Multivariate logistic regression analysis results for severe COVID-19

| **Variables** | **OR（95% CI）** | **P** |
| --- | --- | --- |
| Age,yr | 1.085 (1.047,1.124) | 0.000 |
| BMI/(kg/m2) | 1.156 (1.008,1.324) | 0.038 |
| Fever | 4.612 (1.409,15.102) | 0.012 |
| Sputum production | 3.057 (1.204,7.763) | 0.019 |
| Respiratory rate | 1.650 (1.172,2.322) | 0.004 |
| Pulse oxygen saturation/% | 0.660 (0.517,0.843) | 0.001 |
| NLR | 1.155 (1.043,1.278) | 0.006 |

BMI:body mass index; NLR:neutrophil-to-lymphocyte ratio
