# Supplementary material for: The neutrophil to lymphocyte ratio is an independent predictor for severe COVID-19: Evidence from a multicenter case-control study and meta-analyses
Source: Wien Klin Wochenschr. 2021 Aug 3;133(17-18):882–91. doi: 10.1007/s00508-021-01917-9 (PMC8329905; doi:10.1007/s00508-021-01917-9)
Supplement: Supplementary file 3 — Additional file 3.doc: Newcastle—Ottawa quality assessment scale for case-control study from the meta-analyses [file 508_2021_1917_MOESM3_ESM.doc]

**Newcastle - Ottawa quality assessment scale for case-control study**

**NEWCASTLE - OTTAWA QUALITY ASSESSMENT SCALE**

**CASE CONTROL STUDIES**

Note: A study can be awarded a maximum of one star for each numbered item within the Selection and Exposure categories. A maximum of two stars can be given for Comparability.

**Selection**

1) Is the case definition adequate?

a) yes, with independent validation **🟑**

b) yes, eg record linkage or based on self reports

c) no description

2) Representativeness of the cases

a) consecutive or obviously representative series of cases **🟑**

b) potential for selection biases or not stated

3) Selection of Controls

a) community controls **🟑**

b) hospital controls

c) no description

4) Definition of Controls

a) no history of disease (endpoint) **🟑**

b) no description of source

**Comparability**

1) Comparability of cases and controls on the basis of the design or analysis

a) study controls for _______________ (Select the most important factor.) **🟑**

b) study controls for any additional factor **🟑** (This criteria could be modified to indicate specific control for a second important factor.)

**Exposure**

1) Ascertainment of exposure

a) secure record (eg surgical records) **🟑**

b) structured interview where blind to case/control status **🟑**

c) interview not blinded to case/control status

d) written self report or medical record only

e) no description

2) Same method of ascertainment for cases and controls

a) yes **🟑**

b) no

3) Non-Response rate

a) same rate for both groups **🟑**

b) non respondents described

c) rate different and no designation

The detailed NOS scores of included studies

| First author | NEWCASTLE - OTTAWA QUALITY ASSESSMENT SCALE | | | | | | | | |
| --- | --- | --- | --- | --- | --- | --- | --- | --- | --- |
|  | Selection | | | | Comparabilitiy | Outcome | | | Total |
|  | Q1 | Q2 | Q3 | Q4 | Q1 | Q1 | Q2 | Q3 |  |
| Chuan Qin | 1 | 1 | 1 | 0 | 2 | 1 | 1 | 0 | 7 |
| Jiao Gong | 1 | 1 | 1 | 0 | 2 | 1 | 1 | 0 | 7 |
| Jingyuan Liu | 1 | 1 | 1 | 0 | 2 | 1 | 1 | 0 | 7 |
| Aiping Yang | 1 | 1 | 1 | 0 | 2 | 1 | 1 | 0 | 7 |
| Yafei Zhang | 1 | 1 | 1 | 0 | 2 | 1 | 1 | 0 | 7 |
| Yabing Guo | 1 | 1 | 1 | 0 | 2 | 1 | 1 | 0 | 7 |
| Yabing Guo | 1 | 1 | 1 | 0 | 2 | 1 | 1 | 0 | 7 |
| Zhichao Feng | 1 | 1 | 1 | 0 | 2 | 1 | 1 | 0 | 7 |
| Xu Chen | 1 | 1 | 1 | 0 | 2 | 1 | 1 | 0 | 7 |
| Yongyan Wang | 1 | 1 | 1 | 0 | 2 | 1 | 1 | 0 | 7 |
| Huang Yaxiong | 1 | 1 | 1 | 0 | 2 | 1 | 1 | 0 | 7 |
| Xintian Xia | 1 | 1 | 1 | 0 | 2 | 1 | 1 | 0 | 7 |
| Xi Chen | 1 | 1 | 1 | 0 | 2 | 1 | 1 | 0 | 7 |
| Yidong Pan | 1 | 1 | 1 | 0 | 2 | 1 | 1 | 0 | 7 |
| Wenhua Liang | 1 | 1 | 1 | 0 | 2 | 1 | 1 | 0 | 7 |
| Wenhua Liang | 1 | 1 | 1 | 0 | 2 | 1 | 1 | 0 | 7 |
| Ewan Carr | 1 | 1 | 1 | 0 | 2 | 1 | 1 | 0 | 7 |
| Shaoping Huang | 1 | 1 | 1 | 0 | 2 | 1 | 1 | 0 | 7 |
| Jiangli Cheng | 1 | 1 | 1 | 0 | 2 | 1 | 1 | 0 | 7 |
|  |  |  |  |  |  |  |  |  |  |

Q:question.
