# Supplementary figures and images for: The neutrophil to lymphocyte ratio is an independent predictor for severe COVID-19: Evidence from a multicenter case-control study and meta-analyses
Source: Wien Klin Wochenschr. 2021 Aug 3;133(17-18):882–91. doi: 10.1007/s00508-021-01917-9 (PMC8329905; doi:10.1007/s00508-021-01917-9)

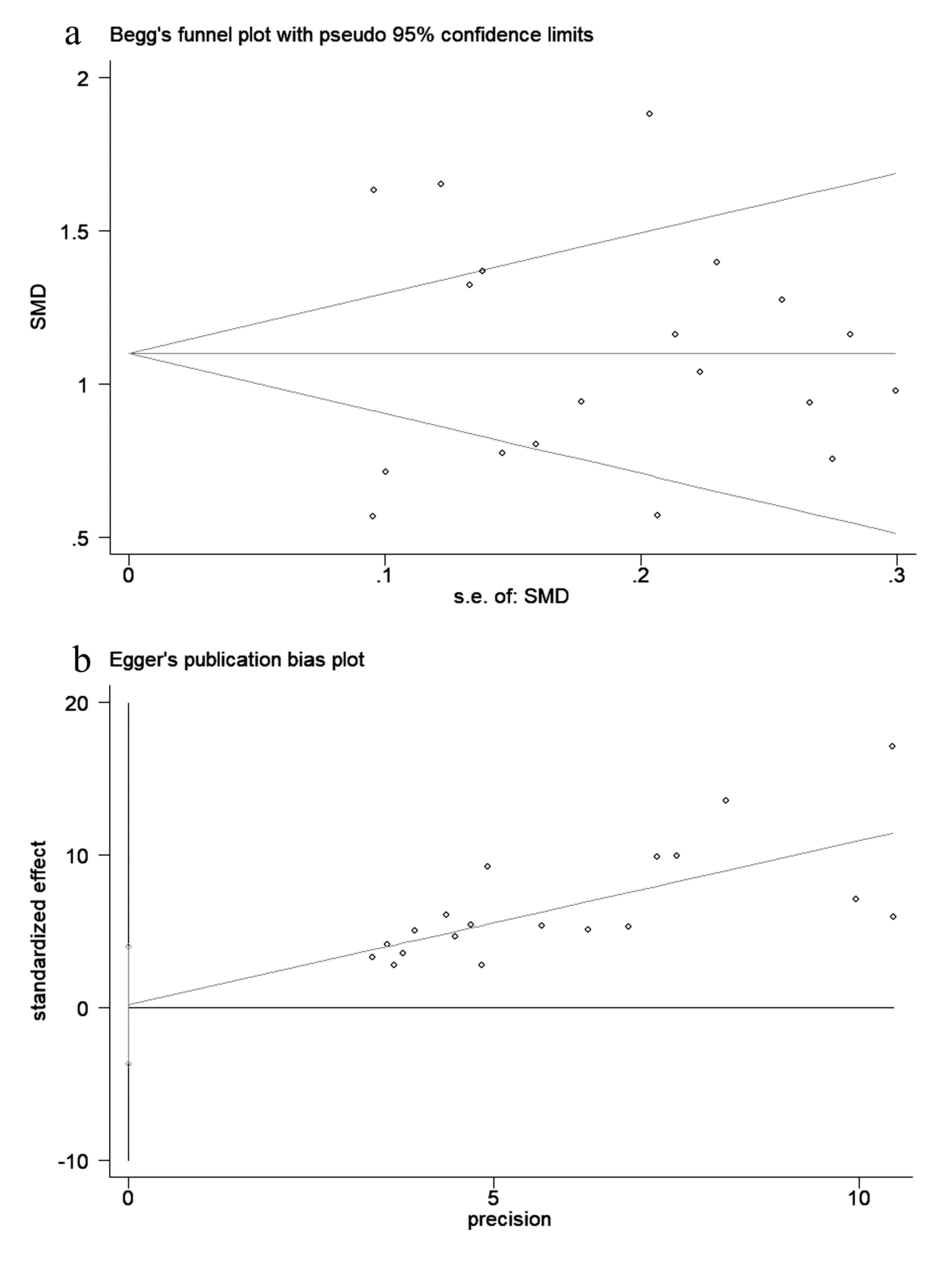

Supplement: Supplementary file 4 — Additional file 4.jpg: Begg’s and Egger’s tests of meta-analyses. [file 508_2021_1917_MOESM4_ESM.jpg]

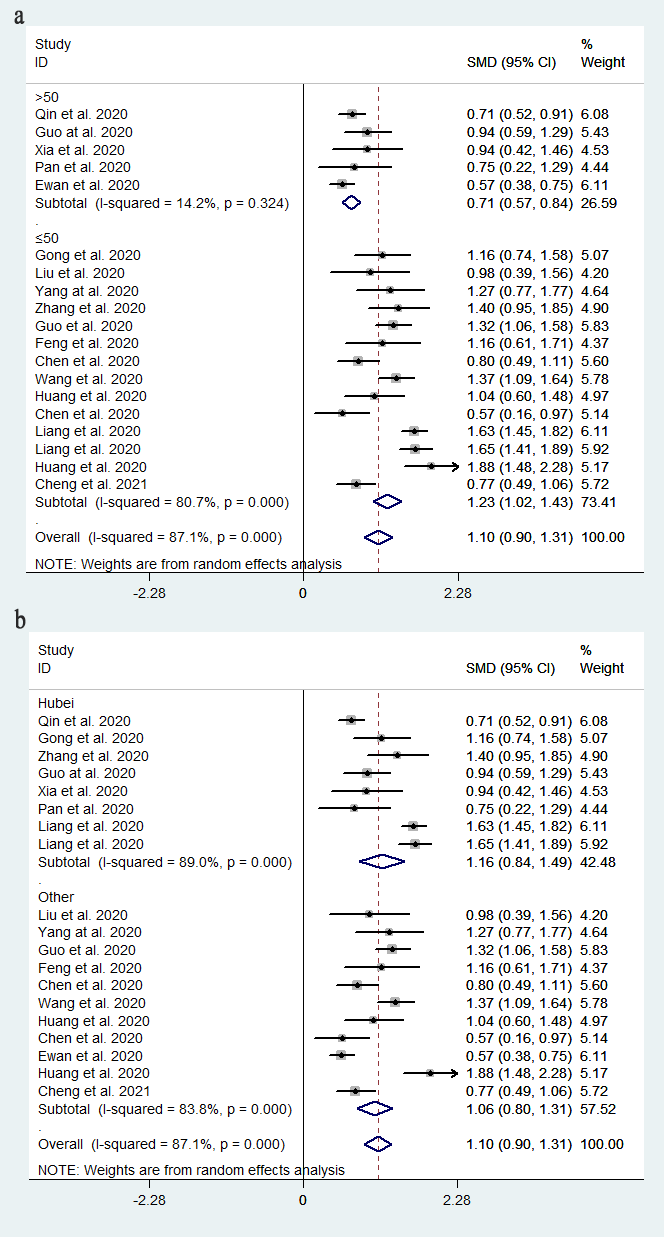

Supplement: Supplementary file 5 — Additional file 5.jpg: The forest plots of subgroup analyses of the meta-analyses. (a) by mean age (> 50 or ≤ 50 years); (b) by study location (Hubei or other). [file 508_2021_1917_MOESM5_ESM.jpg]
