# Supplementary material for: The neutrophil to lymphocyte ratio is an independent predictor for severe COVID-19: Evidence from a multicenter case-control study and meta-analyses
Source: Wien Klin Wochenschr. 2021 Aug 3;133(17-18):882–91. doi: 10.1007/s00508-021-01917-9 (PMC8329905; doi:10.1007/s00508-021-01917-9)
Supplement: Supplementary file 6 — Additional file 6.doc: The 2 × 2 data of NLR in predicting severe COVID-19 of the meta-analyses [file 508_2021_1917_MOESM6_ESM.doc]

The 2×2 data of NLR in predicting severe COVID-19

| Ariticles | TP | FP | FN | TN | ^a^N | ^b^Cut-off |
| --- | --- | --- | --- | --- | --- | --- |
| Yang et al. 2020 | 21 | 25 | 3 | 44 | 93 | 3.3 |
| Chen et al. 2020 | 26 | 52 | 24 | 177 | 279 | 3.5 |
| Wang et al. 2020 | 39 | 136 | 24 | 436 | 635 | 4.06 |
| Xia et al. 2020 | 26 | 8 | 5 | 24 | 63 | 4.795 |
| Huang et al. 2020 | 21 | 97 | 8 | 289 | 415 | 3.5 |
| Cheng et al. 2020 | 53 | 31 | 28 | 101 | 213 | 4.184 |

TP: true positive；FP: false positive；FN: false negative；TN: true negative

^a^N: the case of total people in each article

^b^Cut-off: the cut-off of NLR used in each article
