# Supplementary material for: The neutrophil to lymphocyte ratio is an independent predictor for severe COVID-19: Evidence from a multicenter case-control study and meta-analyses
Source: Wien Klin Wochenschr. 2021 Aug 3;133(17-18):882–91. doi: 10.1007/s00508-021-01917-9 (PMC8329905; doi:10.1007/s00508-021-01917-9)
Supplement: Supplementary file 7 — Additional file 7.doc: The information of hospitals and investigators participated in the case-control study. [file 508_2021_1917_MOESM7_ESM.doc]

**The information of hospitals and investigators participated in the case-control study**

1. Department of Critical Care Medicine (Yan Kang, Xuelian Liao, Bo Wang, Xiaodong Jin, Zhongwei Zhang, Xiaoqi Xie, Wanhong Yin, Chang Liu, Jie Yang, Yongming Tian, Huan Liu, Xiaojin Li, Jun Guo, Min He, Qin Wu, Jin Yang, Aiping Du, Meiling Dong, Jiangli Cheng, Aijia Ma, Luping Wang, Yue Zhou, Chengyong Ma, Xuewei Yang, Jianbo Li, Yan Zhang, Peng Wang, Wei Zhang, Xiangrong Song, Wenxi Xia, Baixu Chen); Department of Respiratory and Critical Care Medicine (Weimin Li, Jun Xiao); Department of Orthopedics (Rui Shi); Department of Clinical Research Management (Xia Zou); Department of Medical Affairs (Bingxing Shuai); Chinese Evidence-based Medicine Center and Cochrane China Center (Wen Wang); West China Hospital, Sichuan University, Chengdu
2. Department of Critical Care Medicine(Hong Chen, Qing Du), The Public Health Clinical Center of Chengdu, Chengdu
3. Clinical Research Center (Zhen li), Shanghai First Maternity and Infant Hospital, Tongji University School of Medicine, Shanghai
4. Department of Infectious Disease (Xiaocui Wu); Department of Critical Care Medicine (Jian Wang, Jin Tang); Guang’an people’s hospital, Guang’an
5. Department of Infectious Disease and Critical Care Medicine (Rui Shi, Yi Xu, Kangjun Ren, Qi Wang); Department of Respiratory and Critical Care Medicine (Jun Xiao); Ganzi Hospital of West China Hospital, Ganzi Tibetan Autonomous Prefeture People’s Hospital, Ganzi
6. Department of Critical Care Medicine (Xiangde Zhen, Wenlai Zhou, Chun Liu), Dazhou Central Hospital, Dazhou
7. Department of Critical Care Medicine (Maojuan Wang, Zhixuan Wu), People's Hospital of Deyang City, Deyang
8. Department of Critical Care Medicine (Xianying Lei, Huan Wang), Affiliated Luzhou Infectious Diseases Hospital of Southwest Medical University, Luzhou
9. Department of Critical Care Medicine (Hongtao Xia, Yu Gong, Xiaoyong Hu), Suining Central Hospital,Suining
10. Department of Critical Care Medicine (Sheng Lv, Hua Zhao), Panzhihua Central Hospital, Panzhihua
11. Department of Critical Care Medicine (Sheng Lv), People’s Hospital of Yanyuan, Liangshan Yi Autonomous Prefecture, Liang shan
12. Department of Critical Care Medicine (Chao Jia, Jun Mo) , Mianyang Central Hospital, Mianyang
13. Department of Critical Care Medicine (Li Chen, Li Zhang), Affiliated Hospital of North Sichuan Medical College, Nan Chong
14. Department of Critical Care Medicine (Juan Shang), Nanchong Central Hospital, Nanchong
15. Department of Critical Care Medicine (Min Yang, Mingxin Xu), People's hospital of ya'an, Ya’an
16. Pulmonary and Critical care medicine (Hailong Wei); Department of Infectious Disease (Jiameng Li); The People’s Hospital of Leshan, Leshan
17. Department of Critical Care Medicine (Huaqiang Shen, Longwen Wang), Bazhong Central Hospital, Bazhong
18. Department of Critical Care Medicine (Xiong Yang), Langzhong People’s Hospital, Langzhong
19. Department of Critical Care Medicine (Xianhua Xiao, Li Li), The Second People’s Hospital of Neijiang, Neijiang
20. Department of Critical Care Medicine (Yuanjun Zhang, Lankai Liao), The first People’s Hospital of Ziyang, Ziyang
21. Department of Critical Care Medicine (Chaogui Zhang, Guiqiang Jin), Yibin Second People’s Hospital of Yibin, Yibin
22. Department of Critical Care Medicine (Minglin Deng, Yanping Li), The first people’s hospital of Liangshan Yi Autonomous Prefecture, Xichang
